# High-throughput muscle fiber typing from RNA sequencing data

**DOI:** 10.1186/s13395-022-00299-4

**Published:** 2022-07-02

**Authors:** Nikolay Oskolkov, Malgorzata Santel, Hemang M. Parikh, Ola Ekström, Gray J. Camp, Eri Miyamoto-Mikami, Kristoffer Ström, Bilal Ahmad Mir, Dmytro Kryvokhyzha, Mikko Lehtovirta, Hiroyuki Kobayashi, Ryo Kakigi, Hisashi Naito, Karl-Fredrik Eriksson, Björn Nystedt, Noriyuki Fuku, Barbara Treutlein, Svante Pääbo, Ola Hansson

**Affiliations:** 1 Department of Clinical Sciences, Lund University, Malmö, Sweden; 2 Department of Biology, Science for Life Laboratory, National Bioinformatics Infrastructure Sweden, Lund University, Lund, Sweden; 3 Max Planck Institute for Evolutionary Anthropology, Leipzig, Germany; 4 Health Informatics Institute, Morsani College of Medicine, University of South Florida, Gainesville, USA; 5 Graduate School of Health and Sports Science, Juntendo University, Chiba, Japan; 6 Swedish Winter Sports Research Centre, Mid Sweden University, Östersund, Sweden; 7 Institute for Molecular Medicine Finland (FIMM), Helsinki University, Helsinki, Finland; 8 Mito Medical Center, Tsukuba University Hospital, Ibaraki, Japan; 9 Faculty of Management & Information Science, Josai International University, Chiba, Japan; 10 Department of Cell and Molecular Biology, Science for Life Laboratory, National Bioinformatics Infrastructure Sweden, Uppsala University, Uppsala, Sweden; 11 Okinawa Institute of Science and Technology, Onna-son, Japan

## Abstract

**Background:**

Skeletal muscle fiber type distribution has implications for human health, muscle function, and performance. This knowledge has been gathered using labor-intensive and costly methodology that limited these studies. Here, we present a method based on muscle tissue RNA sequencing data (totRNAseq) to estimate the distribution of skeletal muscle fiber types from frozen human samples, allowing for a larger number of individuals to be tested.

**Methods:**

By using single-nuclei RNA sequencing (snRNAseq) data as a reference, cluster expression signatures were produced by averaging gene expression of cluster gene markers and then applying these to totRNAseq data and inferring muscle fiber nuclei type via linear matrix decomposition. This estimate was then compared with fiber type distribution measured by ATPase staining or myosin heavy chain protein isoform distribution of 62 muscle samples in two independent cohorts (*n* = 39 and 22).

**Results:**

The correlation between the sequencing-based method and the other two were r_ATPas_ = 0.44 [0.13–0.67], [95% CI], and r_myosin_ = 0.83 [0.61–0.93], with *p* = 5.70 × 10^–3^ and 2.00 × 10^–6^, respectively. The deconvolution inference of fiber type composition was accurate even for very low totRNAseq sequencing depths, i.e., down to an average of ~ 10,000 paired-end reads.

**Conclusions:**

This new method (https://github.com/OlaHanssonLab/PredictFiberType) consequently allows for measurement of fiber type distribution of a larger number of samples using totRNAseq in a cost and labor-efficient way. It is now feasible to study the association between fiber type distribution and e.g. health outcomes in large well-powered studies.

**Supplementary Information:**

The online version contains supplementary material available at 10.1186/s13395-022-00299-4.

## Introduction

Our bodies constitute to ~ 30–40% of the skeletal muscle, and it is the most abundant form of the three types of muscle, the others being smooth and cardiac. The skeletal muscle is composed of different fiber types (i.e., muscle cell types), and the relative proportions of these types vary among the muscles, locations within the muscles, individuals, and the sex of individuals [1–4]. The oxidative and glycolytic potential and the contractile properties differ considerably between fiber types, with the mitochondria-rich slow-twitch fibers (type I) having higher oxidative capacity, and fast-twitch fibers (type IIa and type IIx) having higher glycolytic capacity [1]. The proportions also change as people age, with type II fibers being preferentially affected by sarcopenia [5]. Exercising the skeletal muscle is a major site for catabolic metabolism of the blood glucose and lipids and the metabolic characteristics of this tissue influence both the performance of elite athletes [6, 7] and an individual’s predisposition to disease, e.g., impairments in glucose and lipid transportation into myocytes can promote diabetes and atherosclerotic vascular disease [8–10]. Although environmental factors, such a training or sedentary behavior and aging, lead to adaptive alteration in capillary density and fiber composition (an increase in type IIa vs type IIx) in the skeletal muscle, these features are partially under genetic control [11, 12]. However, the extent to which transition of type I to type II fibers and vice-versa occurs remains uncertain [6]. The muscle tissues are usually classified according to the predominant myosin heavy chain (MyHC) isoforms, but this is a simplified classification that disregards the large number of other proteins expressed in the skeletal muscle [2, 13]. It is thus not straightforward to estimate and compare muscle fiber types in humans, e.g., owing to large heterogeneity and limitations of the methodology, and it is even more challenging in frozen, postmortem samples.

In addition to cells from differing types of muscle fibers, a biopsy sample also contain cells such as fibroblasts, endothelial cells, adipocytes, smooth muscle cells, and neuron-associated Schwann cells. Gene expression analysis of the skeletal muscle is thus rendered difficult by the complexity of this tissue. Although sequencing of transcriptomes from muscle biopsies may still provide a perspective on functional differences between individuals, targeted RNA sequencing from isolated cells provides the opportunity to reveal differences specific to the different cell populations [14]. Previous studies investigating the skeletal muscle have applied single-cell RNA sequencing (scRNAseq) to extracted mono-nucleated cells [15–20]. These studies have for example described a complex landscape of different cell types, e.g., two different populations of muscle progenitor cells [15], provided detailed knowledge concerning muscle regeneration [19] and muscle disease etiology [20]. A challenge for single-cell genomic studies in the muscle and other solid tissues is to capture cell types that are difficult to isolate in suspension [21], e.g., muscle fibers have not been sequenced in the abovementioned scRNAseq studies. However, a few studies have isolated and sequenced single nuclei including the nuclei from poly-nucleated primary muscle fibers in mice [22–24] and e.g. found that myofiber types predominantly express either slow or one of the fast isoforms of MyHC proteins, while only a small proportion of hybrid fibers can express more than one MyHC [22]. However, no study has investigated poly-nucleated primary muscle fibers from humans.

### Results

Here, we present a method to estimate the proportion of skeletal muscle fiber types using only muscle tissue RNA sequencing (totRNAseq) data that could be used on frozen samples. The method is based on snRNAseq information of one human individual and then evaluated in two independent larger totRNAseq data sets of the human skeletal muscle (the Muscle SATellite cell study, MSAT, *n* = 39 and the Juntendo Muscle Study, JMS, *n* = 23).

#### A novel muscle fiber type prediction model derived from single-nuclei RNA sequencing

**Fig. 1:**
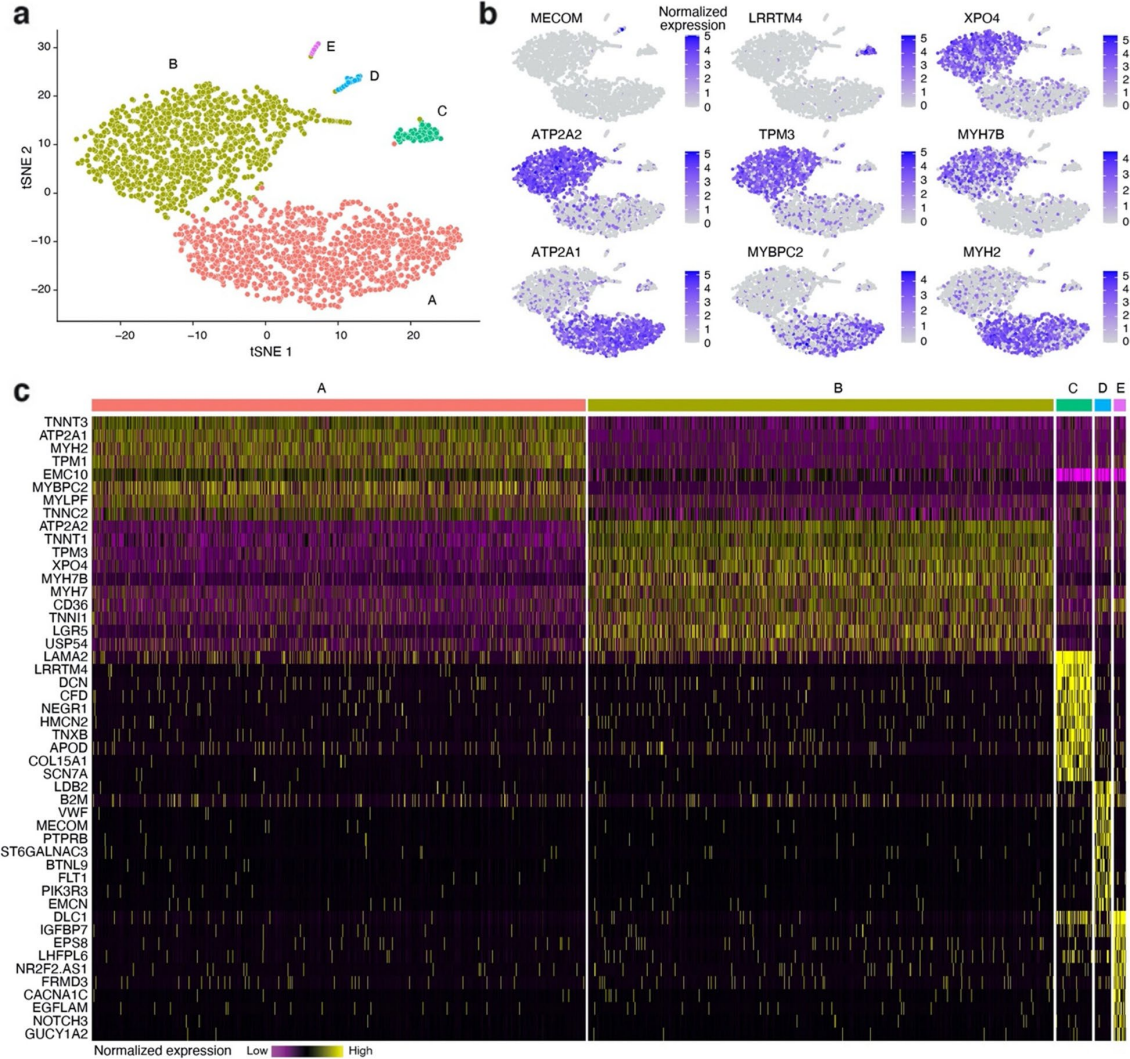
A single-nuclei RNAseq of the human skeletal muscle. Slow- (type I) and fast-twitch (type II) fibers form species-specific distinct clusters of nuclei. **a** Five major clusters of nuclei were identified using graph-based clustering built on Louvain modularity optimization. For visualization of the nuclei populations, t-distributed stochastic neighbor embedding (tSNE) non-linear dimension reduction was applied. **b** Examples of nuclei expression patterns for genes separating different clusters, i.e., *ATP2A1*, *MYBPC2*, and *MYH2* are enriched in cluster A (type II fiber), *XPO4*, *ATP2A2*, *TPM3*, and *MYH7B* are enriched in cluster B (type I fiber), *LRRTM4* is enriched in cluster D (endothelial), and MECOM is enriched in cluster E. **c** Complete list of the 48 marker genes separating the five clusters

After filtering and quality control (see the “Material and methods” for details), the snRNAseq data set consisted of 2699 nuclei, with 22,084 expressed genes. On average, ~ 500 genes were found per nuclei (Supplementary Fig. 1). Five major clusters of nuclei were identified using graph-based clustering built on Louvain modularity optimization [25]. For visualization of the nuclei populations, t-distributed stochastic neighbor embedding (tSNE) non-linear dimension reduction was applied (Fig. 1a). A separation of type I (cluster B) and type II (cluster A) fiber nuclei is clearly observed, i.e., gene markers of type I and type II fibers (e.g.,* ATP2A2*, *TPM3*, *MYH7B* versus *ATP2A1*, *MYBPC2*, *MYH2*) display a distinct expression pattern in different clusters (Fig. 1b). Cluster D is likely representing endothelial cells, i.e., enriched for gene markers like *LDB2*, *VWF*, *BTNL9*, and *FLT1*. The identity of clusters C and E is less clear but could possibly represent fibroblasts respectively pericytes. The complete list of 48 gene markers for the five clusters are shown in Fig. 1c.

#### Muscle fiber typing using total RNA sequencing from frozen samples

**Fig. 2:**
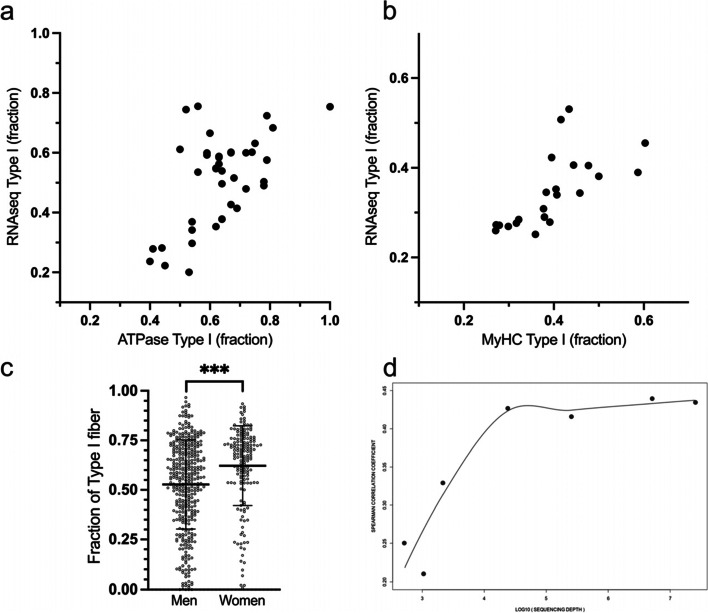
**a** Correlation between the estimated proportions of muscle fiber nuclei types from totRNAseq data and muscle fiber types from ATPase staining in the MSAT study, *r* = 0.44 [0.13–0.67], [95% CI] at *p*_spearman_ = 5.70 × 10^–3^, *n* = 39. **b** Correlation between the estimated proportions of muscle fiber nuclei types from totRNAseq data and muscle fiber types from myosin heavy chain distribution in the JMS study, *r* = 0.83 [0.61–0.93], [95% CI] at *p*_spearman_ = 2.00 × 10^–6^, *n* = 22. **c** Estimated proportions of muscle fiber nuclei types from totRNAseq data from the genotype-tissue expression project (*n*_tot_ = 569). Women had a higher proportion of type I fiber nuclei compared to men, 68% [54–76%] versus 56% [39–71%], median [95% CI], *p*_Mann-Whitney_ = 3.1 × 10^–7^, *n*_women_ = 171, and *n*_men_ = 398. **d** Correlations between the estimated proportions of muscle fiber nuclei types from totRNAseq data and muscle fiber types from ATPase staining in the MSAT study at different sequencing depths

To evaluate the efficacy of estimating proportions of type I versus type II fiber nuclei in muscle samples from totRNAseq data, a deconvolution analysis [26] was performed in a data set consisting of 39 human subjects, i.e., the MSAT study (Table 1). Briefly, by using the snRNAseq data as a reference, cluster expression signatures were produced by averaging gene expression of cluster gene markers and then applying these to the totRNA MSAT data set by inferring muscle fiber nuclei type via linear matrix decomposition [26] and then finally compare this estimate with the fiber type proportions measured by ATPase staining of the same muscle samples. The correlation between the estimated proportions of muscle fiber nuclei types from totRNAseq data and muscle fiber types from ATPase staining was *r* = 0.44 [0.13–0.67], [95% CI] at p_spearman_ = 5.70 × 10^–3^, *n* = 39 (Fig. 2a). The same deconvolution analysis was performed on a second dataset consisting of 23 human subjects from Japan, i.e., the JMS (Table 2). The correlation between the estimated proportions of muscle fiber nuclei types from totRNAseq data and muscle fiber types measured by MyHC protein isoform distribution in the JMS was *r* = 0.83 [0.61–0.93], [95% CI] at p_spearman_ = 2.00 × 10^–6^, *n* = 22 (Fig. 2b). One individual was excluded due to low sequencing quality. To further validate the method skeletal muscle totRNAseq data was downloaded from the Genotype-Tissue Expression project (*n* = 569) and tested for differences in fiber type distribution between men and women. As expected, we observed a larger proportion of type I fibers in women compared to men (68% [54–76%] versus 56% [39–71%], median [95% CI], *p*_Mann-Whitney_ = 3.1 × 10^–7^, *n*_women_ = 171, and *n*_men_ = 398, Fig. 2c).

**Table 1:** Description of the Muscle SATellite cell study (MSAT)

	***N***	**Minimum**	**Maximum**	**Mean**	**SD**
Age (years)	39	21	54	36	8
Weight (kg^BW^)	39	70	97	79	6
BMI (kg^BW^/m^2^)	39	19	31	24	3
VO_2peak_ (ml/kg^BW^ per min)	39	31	70	52	8
Peak power (W/kg^BW^)	39	11	16	13	1
Average power (W/kg^BW^	39	8	10	9	1
Type I (%)	35	40	81	62	10
Type II (%)	35	19	60	38	10

Note: Type II fiber types are given as type II = type IIa + type IIx, all male subjects

**Table 2:** Description of the Juntendo Muscle Study (JMS)

	***N***	**Minimum**	**Maximum**	**Mean**	**SD**
Age (years)	23	20	32	23	3
Weight (kg^BW^)	23	50	72	61	6
BMI (kg^BW^/m^2^)	23	17	26	22	2
Type I (%)	23	27	60	40	9
Type II (%)	23	40	73	60	9

Note: Type II fiber types are given as type II = type IIa + type IIx, 10 female and 13 male subjects

To test the possibility of implementing this method for fiber typing of a large number of samples, we estimated the needed minimal sequencing depth of totRNAseq data that accurately would infer skeletal muscle fiber type composition. An increasing number of randomly selected reads were removed from the totRNAseq data of the 39 samples in the MSAT study. The initial average sequencing depth of 35 million paired-end (PE) reads were “down-sampled” by this method, and Spearman correlations (Fig. 2d) and mean square errors (Supplementary Fig. 2) between the ATPase and totRNAseq predicted fractions of type I fibers were calculated. The accuracy of deconvolution inference of fiber type composition was similar to the 35 million PE reads level even at very low sequencing depths, i.e., down to an of average ~ 10,000 PE reads (Fig. 2d and Supplementary Fig. 2).

## Discussion

Here, we present a method to estimate the proportion of skeletal muscle fiber types from frozen samples allowing for a larger number of samples to be measured in a standardized, cost, and labor-efficient way. Skeletal muscle fiber type distribution is a determinant of physical performance [27–30] and overall health [31–35] and is highly heritable in humans [11, 12]. For example, a reduced proportion of oxidative slow-twitch type I fibers is associated with lower insulin sensitivity in the diabetic muscle [31–34] and muscle atrophy, e.g., age-related sarcopenia is progressing in a fiber type-specific manner [35]. Recently, it has also been shown that skeletal muscle response and recovery from exercise training is dependent on fiber type composition and is thus an important factor to consider in the development of individualized training advice [36–38].

However, many of the abovementioned associations with fiber type distribution are based on low-powered studies. One of the limiting factors is that the methodology for determining fiber type distribution is labor-intensive and thus relatively expensive. The method presented here allows for a larger number of samples to be analyzed using totRNAseq in a standardized and relatively fast way. With this new method, the distribution of type I versus type II fibers can be estimated, but it cannot distinguish between type IIa and type IIx fibers. However, this is achievable with only a small amount of muscle tissue (~ 5–10 mg), e.g., from sampling with the minimally invasive microbiopsy technique [39]. The deconvolution inference of fiber type composition was accurate even for very low sequencing depths, i.e., down to an average of ~ 10,000 PE reads. This means that a shallow-coverage totRNAseq experiment (or targeted RNAseq) will be sufficient to accurately estimate skeletal muscle fiber type composition at a low cost per sample (< 1 dollar/sample). This new method consequently allows for the measurement of fiber type distribution of a larger number of samples and makes it feasible to study the connection between fiber type distribution and health with well-powered studies. It can also be used for estimating fiber type distribution in public repositories of totRNAseq data (e.g., Genotype-Tissue Expression project) and perform in silico analyses of fiber type associations. In conclusion, totRNAseq can efficiently be used to estimate skeletal muscle fiber type distributions of frozen samples.

### Materials and methods

#### snRNAseq data generation

For nuclei isolation from frozen tissue, all following steps were performed on ice with precooled buffers and centrifugation steps were performed at 4 °C. The tissue was disrupted and nuclei liberated through dounce homogenization in ice-cold homogenization buffer (0.32 M sucrose, 3 mM CaCl2, 2 mM magnesium acetate, 0.1 mM EDTA, 1 mM DTT, 10 mg/ml BSA, 10 mM Tris–HCL, protease inhibitors (Sigma-Aldrich)) in the presence of 0.1% NP40. The nuclei suspensions were sequentially passed through 40-, 30-, and 20-μm cell filters (Miltenyi Biotec) and centrifuged at 1000 g for 10 min. The pellet was resuspended in 1% RNase-free BSA in PBS and stained using DAPI (1:1000, BD Pharmingen). Intact single nuclei were sorted in bulk using the DAPI-positive event population, at single-cell sort precision and using a 100-µm nozzle (BD FACS AriaIII) into PBS/1% BSA. Nuclei were counted and loaded on a 10 × chromium microfluidic chip, aiming for the maximum possible number of nuclei to be targeted obtained from the sorting. Single-nucleus experiments were performed using the 10 × genomics single cell 3′ kit v.2 to encapsulate nuclei along with barcode tagged beads, generate, and amplify cDNA and to generate sequencing libraries. Each pooled library was barcoded using i7 barcodes provided by 10 × Genomics. cDNA and sequencing library quality and quantity were determined using Agilent’s High Sensitivity DNA Assay. Libraries were pooled and sequenced in 150-bp paired-end mode on Illumina’s HiSeq platform.

### snRNAseq data processing and analysis

Post-processing pipeline Cell Ranger (https://support.10xgenomics.com/single-cell-gene-expression/software/) provided by 10X Genomics was used for demultiplexing, alignment, filtering, barcode, and unique molecular identifier (UMI) counting. The pipeline produced files in FASTQ and BAM formats, as well as the matrix of UMI counts. We used Seurat workflow for further quality control and downstream analysis of the snRNAseq gene expression data. The initial data set contained 2937 nuclei and 22,336 expressed genes. Nuclei with a high fraction of their counts coming from mitochondrial and ribosomal genes were removed. Next, genes with at least one UMI count present in at least one nucleus were selected. After these steps, 2699 nuclei and 22,084 genes were included in the downstream analysis. We normalized the gene expression data with the LogNormalize method of Seurat and standardized the count values prior to performing the Principal Component Analysis (Supplementary Fig. 3). JackStraw procedure [40] was applied as a denoising step in order to select an optimal number of Principal Components (PCs), indicating that 3 PCs to keep for further downstream analysis, which can be viewed as identifying the true intrinsic dimensionality of the snRNAseq data. Further, the number of cells predicted to be proliferative was investigated using a list of the genes annotated as functioning in the cell cycle according to [41]. The vast majority of cells were not detected to be proliferating and the cycling cells did not form any separate cluster (Supplementary Fig. 4a-b). Graph-based clustering based on Louvain modularity optimization [25] with resolution parameter equal to 0.1 was used for detecting boundaries between different populations of nuclei, and tSNE non-linear dimension reduction with perplexity 50, that was chosen as a square root of the number of nuclei according to the k-nearest neighbors rule of thumb, was applied for visualization of the nuclei populations (Fig. 1a). The snRNAseq data was uploaded to Gene Expression Omnibus (GEO) and is available under accession number GSE190489.

### Deconvolution analysis

Deconvolution analysis [26] was performed using the snRNAseq data as a reference. For this purpose, the snRNAseq data was used to produce cluster signatures, which are marker gene expressions averaged across cells from each cluster. After that, the gene expression of each type of the skeletal muscle fibers in a bulk RNAseq sample was inferred via linear matrix decomposition [42]. The deconvolution analysis was performed using DeconRNASeq R/Bioconductor package, and the results are presented in Fig. 2a, b.

### Study subjects

The MSAT cohort: 39 Swedish male subjects (Table 1) were enrolled in the study by advertising on social media and through local cycling clubs. Inclusion criteria were as follows: (1) male, (2) healthy, not on any medications, and (3) age range between 20 and 55. Subjects were given both oral and written information about the experimental procedures before giving their written informed consent. Each participant went through three visits at different time points. All subjects completed all three visits. The first visit involved a regular doctor’s examination with blood samples and measuring anthropometric characteristics. The second visit consisted, after an overnight fast, of a Wingate test followed by muscle biopsy and VO_2max_ was measured during the third and last visit. The study was approved by the local Ethics committee, Lund University (Dnr 2015/593). For determination of peak anaerobic power (Wingate) and VO_2max_, subjects were instructed to perform only easy training 48 h prior to each test. To determine peak anaerobic power, a 30-s all-out Wingate test [43] was conducted on a cycle ergometer (Monark Peak power). Before the test, a 5-min low intensity ~ 150w warm-up, with instructions to perform a 5 s high cadence drill each minute was performed. The test started with the subject pedaling as fast as possible. When a cadence of 120 rpm was reached, a braking resistance equivalent to 0.7 N × m × kg^−1^ was applied to the freewheel and remained constant during the 30 s. Subjects were instructed to sit down throughout the test. Strong verbal encouragement was given throughout to ensure a maximal effort was provided. An incremental test to exhaustion was performed to determine VO_2max_. The test started with 3 min of cycling at 3 W × kg^−1^ (rounded down to nearest 10 W) and then increased by 35 W every 2 min until voluntary exhaustion or failure to maintain ≥ 60 rpm. Strong verbal encouragement was given throughout. VO_2_ was measured using an Oxycon Pro (Jaeger GmbH, Germany) with a mixing chamber and a 30 s sampling time. Gas sensors were calibrated according to instructions by the vendor before every test. Maximal oxygen uptake was determined as the mean of the two highest values attained during exercise from any 30-s period.

The Juntendo cohort: 23 Japanese subjects (Table 2) were recruited to examine the associations between RNA expression profiles and muscle fiber composition in the Japanese population. All subjects gave their signed informed consent before inclusion in the study. The study protocols were approved by the Ethics Committees of Juntendo University and were performed in accordance with the Declaration of Helsinki. No additional tests were performed in this cohort.

### Muscle biopsies and histology

In the MSAT cohort (Table 1), muscle biopsies were taken from the *vastus lateralis* muscle under sterile conditions and local anesthesia (1% lidocaine) by using a 5-mm Bergström needle and frozen in liquid nitrogen. The biopsies were taken within 5 min after the Wingate test. Serial Sects. (10 μm) were cut using a cryostat at − 20 °C. Myofibrillar ATPase histochemistry was performed by preincubation at pH 4.4, 4.6, and 10.3 to identify fiber types [44]; the proportion of fiber types (i.e., type I, IIa, or IIx) were calculated as the number of each fiber type, divided by the total number of fibers in the section. Computer image analysis was performed using image analysis equipment (BioPix IQ 2.0.16 software, BioPix AB, Sweden).

In the Juntendo cohort (Table 2), muscle biopsies were taken from the *vastus lateralis* muscle under sterile conditions and local anesthesia (1% lidocaine) by using a disposal needle biopsy instrument (Max Core; C. R. Bard, Covington, GA). The biopsies were collected from approximately 15 cm above the patella in both legs of each subject under ultrasound imaging (Noblus; Aloka, Tokyo, Japan) and avoided the inclusion of subcutaneous fat and the subfascial and myotendinous parts as far as possible. In addition, any visible non-muscle tissues (e.g., adipose tissue) were removed from the biopsy samples. Samples were frozen immediately in liquid nitrogen and stored at − 80℃ until further analysis. Myosin heavy chain (MyHC) protein isoforms were assessed as markers of muscle fiber composition. Frozen muscle samples were homogenized in ice-cold lysis buffer [50 mM HEPES (pH 7.4), 10 mM EDTA, 4 mM EGTA, 50 mM β-glycerophosphate, 25 mM NaF, 5 mM Na_3_VO_4_], containing a phosphatase inhibitor (PhosSTOP tablet; Roche Diagnostics, Indianapolis, IN), and a protease inhibitor (Complete tablet; Roche Diagnostics). The lysates obtained were centrifuged at 10,000 g for 10 min at 4℃. An insoluble pellet, obtained after centrifugation, was suspended in a sufficient volume of SDS sample buffer [30% glycerol, 5% β-mercaptoethanol, 2.3% SDS, 0.05% bromophenol blue, and 62.5 mM Tris–HCl (pH 6.8)] and boiled at 95℃ for 5 min. MyHC composition was determined by glycerol SDS-PAGE, according to Kakigi et al. [45]. Briefly, protein samples were resolved by performing glycerol SDS-PAGE [stacking gel: 4% acrylamide, 34.7% glycerol, and 125 mM Tris–HCl (pH 6.8); separating gel: 8% acrylamide, 33.3% glycerol, and 375 mM Tris–HCl (pH 8.3)]. Electrophoresis was started at 60 V with stacking gel at 8℃. The voltage was set to 150 V and run for 18 h at 8℃ when the tracking dye had entered the separating gel completely. After separation, the gels were stained with Coomassie brilliant blue (Biosafe G250; Bio-Rad Laboratories, Hercules, CA) and rinsed repeatedly with water. Each gel was scanned using a calibrated densitometer (ChemiDoc Touch Imaging System; Bio-Rad Laboratories), and the relative proportion of MyHC-I, MyHC-IIa, and MyHC-IIx were determined using the calibrated densitometer (ChemiDoc Touch Imaging System) and analytical software (Image Laboratory software version 5.2.1; Bio-Rad Laboratories).

### RNA extraction and totRNAseq analysis

In the MSAT cohort, RNA was extracted from 25 to 30 mg of muscle biopsies using a TissueLyser II (Qiagen) and the miRneasy Mini Kit (Qiagen). The RNA concentration was determined using a NanoDrop ND-1000 spectrophotometer (A260/A280 > 1.8 and A260/A230 > 1.0 (NanoDrop Technologies, Wilmington, DE, USA). RNA integrity was verified using the 2200 TapeStation instrument (Agilent Technologies, CA, USA), where all samples had an average RNA integrity number (RIN) above 8. In the Juntendo cohort, frozen muscle samples were crushed with 5.0-mm zirconia beads using a Micro Smash MS-100R (Tomy Seiko, Japan) at 3000 rpm twice for 15 s at 2 °C. The total RNA was extracted from muscle samples using TRIzol® Reagent (Thermo Fisher Scientific, Waltham, MA, USA) according to the manufacturer’s protocol. RNA concentration and purity were checked using a NanoDrop 8000 UV–Vis Spectrophotometer (Thermo Fisher Scientific, Wilmington, DE, USA). RNA integrity was verified using the 2200 TapeStation instrument (Agilent Technologies, CA, US), where all samples had an average RNA integrity number (RIN) above 8.

All samples from both the MSAT and Juntendo cohorts were sequenced at Lund University using 800 ng input RNA. Library preparation was made using the TruSeq Stranded Total RNA Library Prep Kit with Ribo-Zero Human/Mouse/Rat Set A (Illumina), and the 75 bp paired-end sequencing was performed on a NextSeq instrument using the NextSeq® 500/550 High Output Kit v2 (150 cycles) (Illumina). The sequencing quality was checked with fastQC v0.11.9 (http://www.bioinformatics.babraham.ac.uk/projects/fastqc) and multiQC [46] v1.9. Gene expression was assessed using Salmon [47] v1.2.1. Exon expression was obtained by mapping reads with STAR [48] v2.7.6 and counting with featureCounts [49] v2.0.1 (options: -p -f -C -O). We used the GRCh38 Ensembl v77 [50] as a reference genome. We used the Seqtk tool (https://github.com/lh3/seqtk) for gradual random downsampling of the MSAT data and applied Salmon for quantifying gene expression of the downsampled RNAseq data. The quantified gene expression for each downsampling iteration was normalized with TMM [51], and deconvolution analysis was performed using the gene markers identified for slow- and fast-twitch human clusters in the snRNAseq data as described above. We computed the Spearman correlation coefficient and mean square deviation between predicted and true fiber type composition for each downsampling iteration.

## Supplementary Information

**Additional file 1: Figure S1.** Seurat snRNAseq workflow quality control statistics. From left to right, first figure reports the number of expressed genes per cell, second figure reports the number of UMIs (library size) per cell, third figure shows the fraction of reads mapped to mitochondrial genes per cell. **Figure S2.** The initial average sequencing depth of 35 million paired-end (PE) reads were ´down-sampled´ and mean square errors between the ATPase and totRNAseq predicted fractions of Type I fibers were calculated. **Figure S3.** Principal Component Analysis (PCA) plot of gene exppression from human skeletal muscle snRNAseq colored by the cluster annotation, see Figure 1A, obtained from the Louvain clustering on the number of significant principal components (according to Seurat workflow). **Figure S4.** (a) Principal Component Analysis (PCA) plot, and (b) tSNE plot of gene expression from human skeletal muscle snRNAseq colored by the cell cycle annotation obtained from the CellCycleScoring function from Seurat workflow. No obvious cluster formation based on the cell cycle can be observed from neither the PCA nor the tSNE plot.

## Data Availability

All scripts needed to utilize this new methodology is openly available at https://github.com/OlaHanssonLab/PredictFiberType. The datasets supporting the conclusions of this article are available in the Gene Expression Omnibus repository, GSE190489 at https://www.ncbi.nlm.nih.gov/geo. The totRNAseq data are available in the European Genome-phenome Archive, EGAS00001006362 - Juntendo Muscle Study (JMS), EGAS00001006363 - Muscle SATellite cell study (MSAT) at https://ega-archive.org.

## Abbreviations

totRNAseq: Muscle tissue RNA sequencing data
scRNAseq: Single-cell RNA sequencing
snRNAseq: Single-nuclei RNA sequencing
Type I: Slow-twitch fibers
Type II: Fast-twitch fibers
MyHC: Myosin heavy chain
MSAT: The muscle SATellite cell study
JMS: Juntendo Muscle Study
tSNE: T-Distributed stochastic neighbor embedding
*ATP2A2*: ATPase sarcoplasmic/endoplasmic reticulum Ca2 + transporting 2
*TPM3*: Tropomyosin 3
*MYH7B*: Myosin heavy chain 7B
*ATP2A1*: ATPase sarcoplasmic/endoplasmic reticulum Ca2 + transporting 1
*TNNT3*: Troponin T3, fast skeletal type
*MYH2*: Myosin heavy chain 2
*TTN*: Titin
*MEG3*: Maternally expressed 3
PE: Paired-end
UMI: Unique molecular identifier
PCs: Principal components
PCA: Principal Components Analysis
GEO: Gene Expression Omnibus
RIN: RNA integrity number
EGA: European Genome-phenome Archive
