# Supplementary material for: High-throughput muscle fiber typing from RNA sequencing data
Source: Skelet Muscle. 2022 Jul 2;12:16. doi: 10.1186/s13395-022-00299-4 (PMC9250227; doi:10.1186/s13395-022-00299-4)
Supplement: Supplementary file 1 — Additional file 1: Figure S1. Seurat snRNAseq workflow quality control statistics. From left to right, first figure reports the number of expressed genes per cell, second figure reports the number of UMIs (library size) per cell, third figure shows the fraction of reads mapped to mitochondrial genes per cell. Figure S2. The initial average sequencing depth of 35 million paired-end (PE) reads were ´down-sampled´ and mean square errors between the ATPase and totRNAseq predicted fractions of Type I fibers were calculated. Figure S3. Principal Component Analysis (PCA) plot of gene exppression from human skeletal muscle snRNAseq colored by the cluster annotation, see Figure 1A, obtained from the Louvain clustering on the number of significant principal components (according to Seurat workflow). Figure S4. (a) Principal Component Analysis (PCA) plot, and (b) tSNE plot of gene expression from human skeletal muscle snRNAseq colored by the cell cycle annotation obtained from the CellCycleScoring function from Seurat workflow. No obvious cluster formation based on the cell cycle can be observed from neither the PCA nor the tSNE plot. [file 13395_2022_299_MOESM1_ESM.docx]

**Supplementary figures**


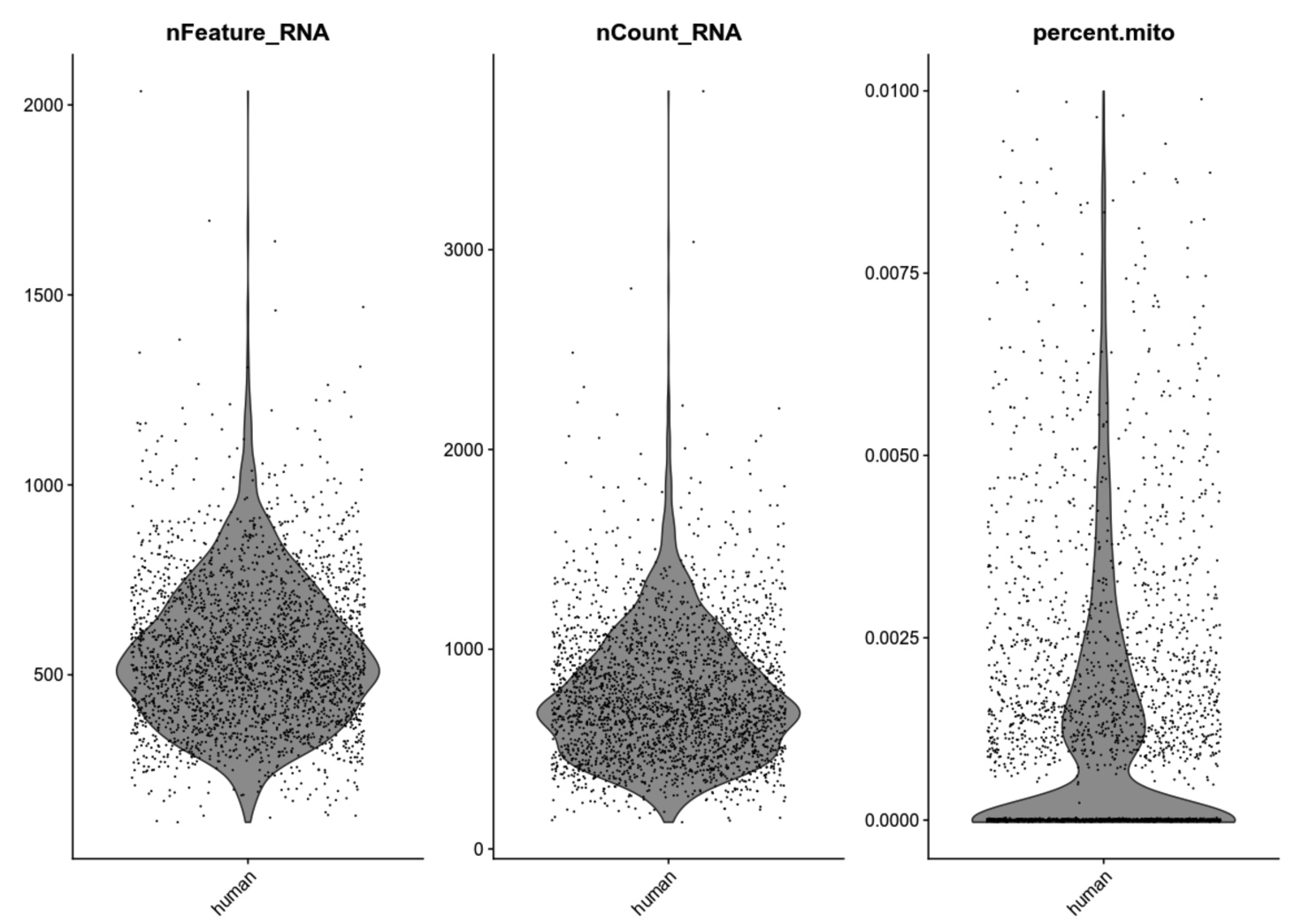


**Supplementary figure 1.**

Seurat snRNAseq workflow quality control statistics. From left to right, first figure reports the number of expressed genes per cell, second figure reports the number of UMIs (library size) per cell, third figure shows the fraction of reads mapped to mitochondrial genes per cell.


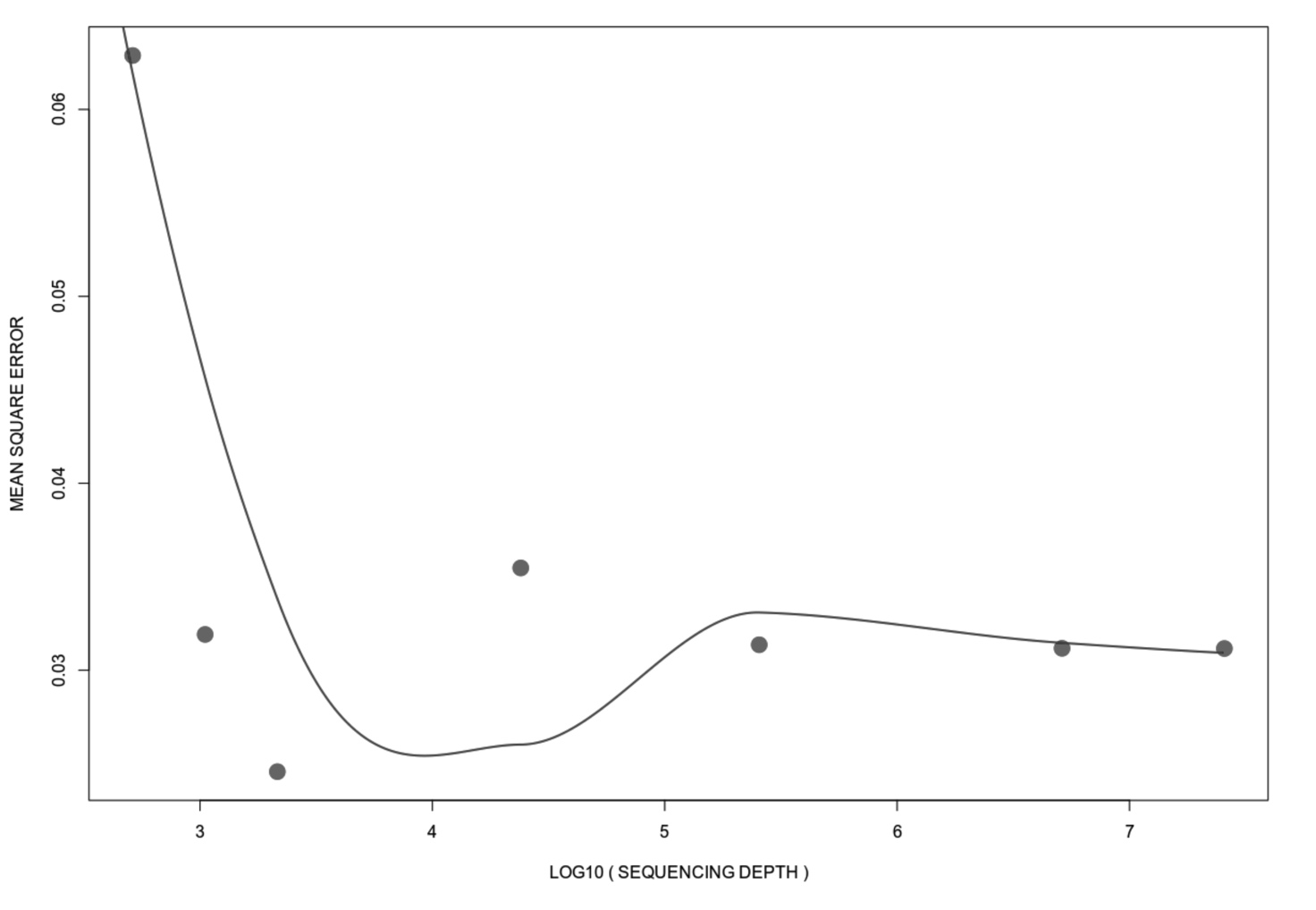


**Supplementary figure 2.** The initial average sequencing depth of 35 million paired-end (PE) reads were ´down-sampled´ and mean square errors between the ATPase and totRNAseq predicted fractions of Type I fibers were calculated.


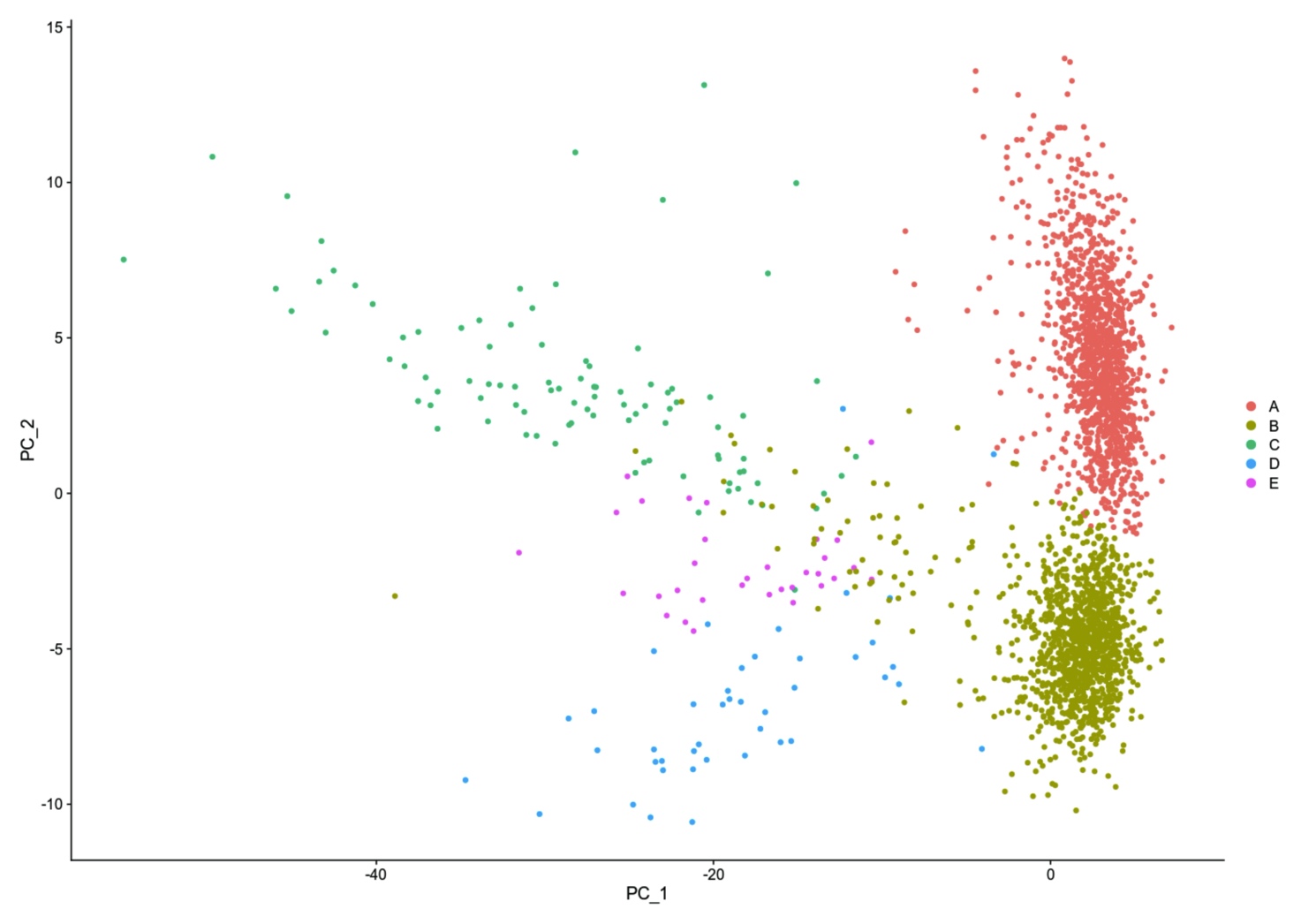


**Supplementary figure 3.** Principal Component Analysis (PCA) plot of gene exppression from human skeletal muscle snRNAseq colored by the cluster annotation, see Figure 1A, obtained from the Louvain clustering on the number of significant principal components (according to Seurat workflow).


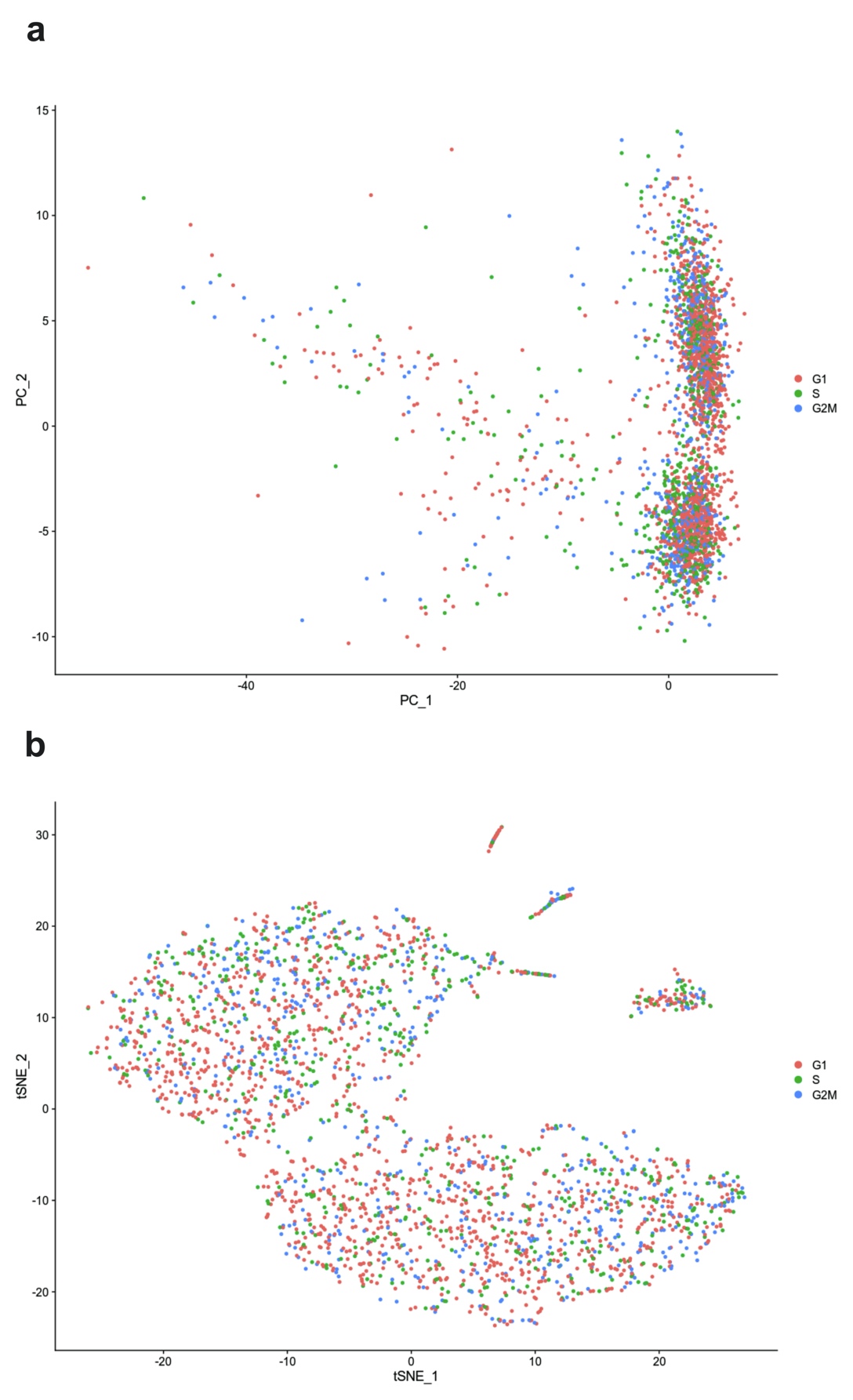


**Supplementary figure 4.** (**a**) Principal Component Analysis (PCA) plot, and (**b**) tSNE plot of gene expression from human skeletal muscle snRNAseq colored by the cell cycle annotation obtained from the CellCycleScoring function from Seurat workflow. No obvious cluster formation based on the cell cycle can be observed from neither the PCA nor the tSNE plot.
